# Mebendazole Exerts Anticancer Activity in Ovarian Cancer Cell Lines via Novel Girdin-Mediated AKT/IKKα/β/NF-κB Signaling Axis

**DOI:** 10.3390/cells14020113

**Published:** 2025-01-14

**Authors:** Rahul Gupta, Dipanjan Roy, Arijit Ghosh, Yasmin Begum, Dipanjan Ghosh, Snehasikta Swarnakar

**Affiliations:** 1 Infectious Diseases & Immunology Division, Council of Scientific and Industrial Research-Indian Institute of Chemical Biology, Jadavpur, Kolkata 700032, India or rahulgupta@csiriicb.res.in (R.G.); or yasmin@csiriicb.res.in (Y.B.); 2 Department of Natural Products, National Institute of Pharmaceutical Education and Research, Kolkata 700054, India; roydipanjan@niperkolkata.ac.in; 3 Department of Molecular Biology, Netaji Subhash Chandra Bose Cancer Research Institute, Kolkata 700094, India; jitbiochem71@gmail.com

**Keywords:** mebendazole, girdin, MMP-9, EMT, ovarian cancer, metastasis

## Abstract

Mebendazole (MBZ), a benzimidazole anthelmintic and cytoskeleton-disrupting compound, exhibits antitumor properties; however, its action on ovarian cancer (OC) is not clearly understood. This study evaluates the effect of MBZ on OC cell lines OVCAR3 and OAW42, focusing on cell proliferation, migration, invasion, and cancer stemness. The underlying mechanisms, including cytoskeletal disruption, epithelial–mesenchymal transition (EMT), and signaling pathways, were explored. MBZ inhibited OVCAR3 and OAW42 cell proliferation in a dose- and time-dependent manner. Additionally, MBZ significantly impedes migration, spheroid invasion, colony formation, and stemness. In addition, it reduced actin polymerization and down-regulated CSC markers (e.g., CD24, CD44, EpCAM). Moreover, MBZ suppressed MMP-9 activity and inhibited the EMT marker as judged by decreased N-Cadherin and Vimentin and increased E-Cadherin. Furthermore, MBZ induced G2/M cell cycle arrest by modulating Cyclin B1, CDC25C, and WEE1. Also, it triggered apoptosis by disrupting mitochondrial membrane potential. Mechanistic studies revealed a significant downregulation of Girdin, an Akt modulator, along with reduced p-Akt, p-IKKα/β, and p-NF-κB, indicating MBZ’s novel mechanism of action through the Girdin-mediated Akt/IKKα/β/NF-κB signaling axis. Thus, by targeting Girdin, MBZ presents a promising repurposed therapeutic strategy to inhibit cancer cell proliferation and metastasis in ovarian cancer.

## 1. Introduction

Ovarian cancer (OC), one of the deadliest gynecological malignancies, is characterized by high mortality rates with few effective treatment options available. Most OCs originate from epithelial cells, comprising the epithelial ovarian cancer subtype, which is the most common form and often spreads within the peritoneal cavity and metastasizes to other parts of the body via the lymphatic system and bloodstream [1]. Early metastasis and frequent recurrence contribute to the poor outcome of the disease. Current standard treatment typically includes cytoreductive surgery to remove the tumor, followed by chemotherapy, with or without radiotherapy, to kill residual cancer cells. OC cells interact dynamically with the surrounding cells and extracellular matrix (ECM) proteins, significantly influencing their metastatic potential. This process involves key factors such as matrix metalloproteinases (MMPs), integrins, Girdin, growth factors, the tumor microenvironment, and epithelial-to-mesenchymal transition (EMT). These factors interact through complex signaling networks to drive cancer progression and metastasis, often accompanied by changes in cell morphology and metabolism [2] Additionally, actin polymerization, ATP generation by mitochondrial activity, and cancer stem cell (CSC) characteristics also play a critical role in enabling these processes. A deeper understanding of these interactions provides valuable insights into potential therapeutic targets to halt cancer progression and metastasis [3]. Furthermore, the functional interplay between Girdin and MMP-9 is critical in cancer progression. Girdin, a cytoskeletal scaffold protein, regulates cancer cell motility, facilitating tissue invasion while MMP-9 remodels the ECM and breaks down barriers to enable metastasis [4,5]. Their combined activity might underscore the intricate molecular network driving cancer progression. Current therapies inadequately address these complex mechanisms, thus innovative treatments targeting both Girdin and MMP-9 are needed to effectively stop metastasis.

Mebendazole (MBZ) is a widely recognized anti-helminthic agent that has shown promising anticancer activity with low toxicity. It has been found to target various signaling pathways implicated in cancer progression, including those related to metastasis and chemoresistance [6]. MBZ’s anticancer effects are primarily attributed to its ability to inhibit tubulin polymerization, resulting in mitotic arrest and promoting apoptosis in cancer cells. Furthermore, MBZ can prevent tumor growth and metastasis in several cancer types, such as lung, breast, and colorectal cancers [7,8,9]. In addition, MBZ has emerged as a potential anticancer agent, with clinical trials demonstrating its efficacy and broad therapeutic potential. For example, in a clinical trial (NCT01729260), MBZ has been evaluated in combination with temozolomide for high-grade gliomas, demonstrating safety and improved survival outcomes [10]. Another clinical trial (NCT03628079) investigating MBZ in advanced malignancies demonstrated its safety and efficacy, particularly in targeting gastrointestinal cancer [11]. Pediatric trials (NCT02644291) are also investigating MBZ for brain cancers, showing its ability to cross the blood–brain barrier, inhibit tumor progression, and assess safety profiles [12].These findings prompted us to explore its significant potential as a repurposed therapeutic agent for OC treatment. Further, elevated Girdin expression has been observed in several cancers, including gastric cancer, where it contributes to tumor progression by activating the Akt/GSK3β/β-catenin signaling pathway [13]. The knockdown of Girdin disrupts the AKT-mediated signaling pathways in several cancers, resulting in the inhibition of proliferation, metastasis, angiogenesis, and induces apoptosis [14,15,16,17]. Moreover, previous studies have demonstrated that MBZ suppresses the PI3K/Akt and NF-κB pathways, both of which are pivotal for cancer cell proliferation, survival, and metastasis. Furthermore, MBZ inhibition of the PI3K/Akt pathway has been shown to sensitize ovarian cancer cells to chemotherapeutic agents like cisplatin [18]. Notably, in oral tongue squamous cell carcinoma (OTSCC), MBZ combined with paclitaxel enhanced the inhibition of PI3K/Akt phosphorylation and induced apoptosis, highlighting its potential to disrupt key oncogenic pathways [19]. However, a direct link between MBZ’s effects on Girdin and its downstream Akt and NF-κB signaling in the context of ovarian cancer has not been previously explored.

Considering the lack of direct evidence linking MBZ’s effects on Girdin to Akt and NF-κB signaling, we designed experiments to elucidate MBZ’s anticancer mechanisms in ovarian cancer (OC) cell lines (OVCAR3 and OAW42). Our findings reveal, for the first time, that MBZ exerts its anticancer effects in OC cells by down-regulating Girdin expression, leading to the inhibition of the Akt/IKKα/β/NF-κB signaling axis. This suppression disrupts actin polymerization, reduces cancer stemness, induces cell cycle arrest, and enhances apoptosis in OC cells. These findings not only establish MBZ’s potential as a therapeutic agent for OC but also identify a novel mechanism targeting Girdin and its downstream signaling cascade, providing new avenues for therapeutic intervention.

## 2. Materials and Methods

### 2.1. Cell Culture Reagents

Cell lines (OVCAR3 and OAW42 ovarian cancer cell lines, procured from Dr. Amit Kumar Srivastava’s lab at CSIR-IICB, Kolkata); RPMI-1640 Medium (Thermo Fisher Scientific, Cat# 11875093, Waltham, MA, USA); Fetal Bovine Serum (FBS) (Thermo Fisher Scientific, Cat# 26140079, Waltham, MA, USA); Penicillin/Streptomycin Solution (100×) (Thermo Fisher Scientific, Cat# 15140122, Waltham, MA, USA); Trypsin-EDTA Solution (Thermo Fisher Scientific, Cat# 25200056, Waltham, MA, USA).

### 2.2. Chemicals

Dimethyl Sulfoxide (DMSO) (Sigma-Aldrich, Cat# D8418, Burlington, MA, USA); Mebendazole (MBZ) (Sigma-Aldrich, Cat# M1625, Burlington, MA, USA); Cisplatin (Sigma-Aldrich, Cat# 232120, USA); MTT Reagent (Thermo Fisher Scientific, Cat# M6494, Waltham, MA, USA); Phosphate-Buffered Saline (PBS) (Thermo Fisher Scientific, Cat# 10010023, Waltham, MA, USA); Paraformaldehyde (Sigma-Aldrich, Cat# 158127, Burlington, MA, USA); Crystal Violet Solution (Sigma-Aldrich, Cat# C6158, Burlington, MA, USA); RIPA Lysis Buffer (Cell Signaling Technology (CST), Cat# 9806, USA); BSA (Sigma-Aldrich, Cat# A3059, Burlington, MA, USA); Triton X-100 (Sigma-Aldrich, Cat# T9284, Burlington, MA, USA); Rhodamine 123 (Thermo Fisher Scientific, Cat# R302, Waltham, MA, USA); Phalloidin-FITC (Sigma-Aldrich, Cat# P5282, Burlington, MA, USA); PVDF Membrane (Merck, Immobilon-P, Cat# IPVH00010, Rahway, NJ, USA); HRP Substrate Immobilon Western Chemiluminescent HRP (Merck, Cat# WBKLS0500, Rahway, NJ, USA); 3 color Prestained Protein Ladder, 10–250 kDa (Genetix Biotech); ProLong Gold antifade reagent with DAPI (Invitrogen, Cat#P36935, Waltham, MA, USA).

### 2.3. Antibodies

E-cadherin (Cat# 3195), N-cadherin (Cat# 13116), Vimentin (Cat# 5741), Cyclin B1 (Cat# 4138), Cdc25C (Cat# 4688), Wee1 (Cat# 4910), MMP-9 (Cat# 2270), Bax (Cat# 2772), Bcl-2 (Cat#2872), Cleaved-PARP (Cat# 9541), Cleaved-Caspase-3 (Cat# 9661), Cleaved-Caspase-9 (Cat# 9501), Cleaved-Caspase-7 (Cat# 9491), Cytochrome C (Cat# 11940), Girdin (Cat# 2629), pAkt (Cat# 4060), p-IKKα/β (Cat# 2697), p-NF-κB (p65) (Cat# 3033), Anti-Rabbit IgG, HRP-linked Antibody (Cat# 7074), Anti-Mouse IgG, HRP-linked Antibody (Cat# 7076), Alexa Fluor 488-Conjugated Anti-Rabbit IgG (Cat# 4412) were purchased from Cell Signaling Technology, CST, USA, GAPDH (Santa Cruz Biotechnology, Dallas, TX, Cat# sc-47724, USA). CD44-PerCP Cy5.5 (BD Biosciences, Cat# 560531, San Jose, CA, USA), CD24-FITC (BD Biosciences, Cat# 555427, San Jose, CA, USA), EpCAM-APC (BD Biosciences, Cat# 347200, San Jose, CA, USA).

### 2.4. Equipments

Humidified incubator (HERAcell 204i, ThermoFisher Scientific, Waltham, MA, USA), Centrifuge 5804R (Thermo Fisher Scientific, Waltham, MA, USA), microplate reader (Thermo Fisher Scientific, Model: Multiskan FC, Waltham, MA, USA), confocal microscope (Olympus, Model: FV3000, Tokyo, Japan), fluorescence microscope (Olympus, Model: IX73, Tokyo, Japan), ChemiDoc Imaging System (Bio-Rad, Model: ChemiDoc MP, Hercules, CA, USA), flow cytometer (BD Biosciences, Model: FACSLyric™, San Jose, CA, USA).

### 2.5. Cell Proliferation Assay

The experiment was performed following established protocol [20], with some modifications. Briefly, OVCAR3 and OAW42 cells (10,000/well) were seeded onto a 96-well plate in RPMI 1640 supplemented with 10% FBS and 1% penicillin/streptomycin, and incubated at 37 °C in 5% CO_2_ overnight. Upon reaching 70–80% confluency, the cells were exposed to varying concentrations of MBZ (0.156, 0.312, 0.625, 1.25, and 2.5 μM), and 0.1% DMSO served as the vehicle control (VC). After treatment, the plates were incubated at 37 °C in 5% CO_2_ for 24, 48, and 72 h. Following the incubation period, the media was aspirated, and MTT solution was added to each well, followed by the addition of DMSO after 3 h of incubation with MTT. Cell viability was assessed by measuring the optical density at 570 nm using a microplate reader. The concentration of MBZ required to inhibit cell growth by 50% (IC_50_) was determined for each time point using the generated cell viability curves analyzed with GraphPad Prism 5 software. Additionally, the concentrations of MBZ required to inhibit cell growth by 25% (IC_25_) and 75% (IC_75_) were calculated at the 48 h time point to establish the dose-dependent response of MBZ for subsequent experiments. In addition to this, the cells were exposed to varying concentrations of Cisplatin (0.156, 0.312, 0.625, 1.25, and 2.5 μM), and 0.1% DMSO served as the vehicle control (VC) for 48 h and the IC_50_ was determined similarly as discussed above. Each experiment was performed in triplicate to ensure reproducibility.

### 2.6. Cell Migration (Scratch Wound Healing) Assay

In accordance with the standard protocol [21], the experiment was carried out with a few alterations. Briefly, a total of 100,000 OVCAR3 and OAW42 cells were seeded per well in 6-well plates containing growth medium and cultured for 24 h to reach 85–90% confluency. A scratch was made on the surface of the cell layer using a sterile pipette tip, creating a wound. The cell culture medium was removed, and the cells were rinsed with phosphate-buffered saline before treatment. Cells were exposed to MBZ at the indicated concentrations [OVCAR3 (0.312, 0.625, and 1.25 µM) and OAW42 (0.156, 0.312, and 0.625 µM] and a DMSO (VC) control for 48 h. Images of cell migration and wound closure were taken at the start of the experiment and 48 h later accordingly using a microscope (magnification 10×, scale bar = 100 μm). The area of cell migration was then measured using ImageJ software and the migration rate was calculated based on the percentage of scratch closure change and the quantitative plots were generated using GraphPad Prism 5 software. Each experiment was conducted at least three times to ensure reproducibility.

### 2.7. 3D Spheroid Invasion Assay

The experimental procedure followed the standard protocol described in references [22,23], with minor adjustments to the cell density and spheroid formation incubation time. A 3D spheroid invasion assay was conducted to evaluate the impact of MBZ on cell invasion. Briefly, the OVCAR3 and OAW42 cells were harvested, and a cell suspension was prepared in RPMI 1640 medium containing 10% FBS. The cell suspension was centrifuged and resuspended in RPMI 1640 with 10% FBS. Then, 20 µL of the cell suspension (2000 cells) was pipetted onto the inside portion of the lid of a non-adhesive 12-well plate. The lid was inverted and placed back onto the 12-well plate containing 1 mL of sterile PBS on each well for humidification, creating hanging drops of the cell suspension. The cells in the hanging drops were incubated at 37 degrees Celsius in 5% CO_2_ for 12 h. After 12 h, 10 µL of fresh RPMI 1640 containing 10% FBS was added to each drop and incubated at 37 degrees Celsius in 5% CO_2_ for an additional 12 h to promote spheroid formation. After spheroid formation, each spheroid was transferred to a new adhesive 12-well plate containing RPMI with 10% FBS and incubated at 37 degrees Celsius in 5% CO_2_ for 12–16 h to adhere. Once adhered to the plate, the spheroids were treated with MBZ at varying concentrations [OVCAR3 (0.312, 0.625, and 1.25 µM) and OAW42 (0.156, 0.312, and 0.625 µM] and a DMSO (VC) for 48 h, and, subsequently, images were taken at the start of the experiment and 48 h later using an inverted microscope with 10× magnification, scale bar = 100 μm. The area of spheroid invasion was then measured using ImageJ 1.5.3 software. The extent of spheroid invasion was determined by subtracting the initial spheroid radius from the final radius of the invaded area and the quantitative plots were generated using GraphPad Prism 5 software. The experiments were repeated at least three times for reliability.

### 2.8. Colony Formation Assay

The experiment was carried out following established protocol [24], with few modifications. Briefly, the OVCAR3 and OAW42 cells were seeded into 6-well plates at a density of 250 cells per well and allowed to adhere for 24 h. Subsequently, the cells were exposed to varying concentrations of MBZ [OVCAR3 (0.312, 0.625, and 1.25 µM) and OAW42 (0.156, 0.312, and 0.625 µM] and a DMSO (VC) control for 48 h. Following treatment, the culture medium was refreshed every 2–3 days. After 10 days, the medium was aspirated, and the cells were rinsed twice with phosphate-buffered saline (PBS). The cells were then fixed with 4% paraformaldehyde for 15 min and stained with a 0.5% crystal violet solution for 30 min. Subsequently, the cells were washed with PBS and air-dried, the number of colonies was manually counted, and the quantitative plots were generated using GraphPad Prism 5 software. Each experiment was independently repeated a minimum of three times.

### 2.9. Assessing Actin Polymerization with Flow Cytometry

Following the standard protocol [25], we performed the experiment with some modifications. Briefly, OVCAR3 and OAW42 cells were seeded at a density of 1 × 10^6^ cells in 6-well plates containing RPMI 1640 supplemented with 10% FBS and 1% penicillin/streptomycin. The cells were then incubated at 37 °C in 5% CO_2_ for 24 h until they reached 80–90% confluency. Following this, the complete media was removed, and the cells were exposed to varying concentrations of MBZ [OVCAR3 (0.312, 0.625, and 1.25 µM) and OAW42 (0.156, 0.312, and 0.625 µM] and a DMSO (VC). Subsequently, the cells were trypsinized, centrifuged at 2000 rpm for 5 min, washed with PBS, and then fixed with paraformaldehyde at a final concentration of 4% and incubated for 15–30 min at room temperature. Cells were then washed twice with PBS to remove excess fixative. Permeabilization buffer (0.1% Triton X-100) was added to the fixed cells and incubated for 10–15 min at room temperature. Cells were then washed again twice with PBS to remove excess permeabilization buffer. Phalloidin-FITC conjugate solution in PBS was added to the cells at a recommended concentration of 50 µg/mL and incubated for 30–60 min at room temperature, protected from light. Cells were washed twice with PBS to remove unbound phalloidin-FITC. Cells were finally resuspended in a flow cytometry buffer and analyzed using BD FACSLyric^TM^ (BD Biosciences, USA). Fluorescence intensity of phalloidin-FITC from different treatment groups, which corresponds to the amount of F-actin in the cells, was collected. The change in fluorescence intensity of different experimental groups was compared to assess the effect of MBZ in actin polymerization. The mean fluorescence intensity (MFI) of different treatment groups was finally analyzed with FlowJo 10 software and the quantitative plots were generated using GraphPad Prism 5 software.

### 2.10. Evaluating Actin Polymerization with Confocal Microscope

The protocol referenced in [26,27], was used to conduct the experiment, with a few modifications. Briefly, both the OC cell lines were cultured as described in previous methods. Subsequently, the culture medium was replaced with IC_50_ concentrations of MBZ solution (OVCAR3: 0.625 μM and OAW42: 0.312 μM) or DMSO (0.1% *v*/*v*) as a vehicle control. Cells were then incubated for an additional 48 h. After 48 h, cells were washed with PBS and then Paraformaldehyde was added to the cells seeded in the coverslips at a final concentration of 4% and incubated for 15–30 min at room temperature. Cells were washed twice with PBS to remove excess fixative. Permeabilization buffer (0.1% Triton X-100) was added to the fixed cells and incubated for 10–15 min at room temperature. Cells were washed twice with PBS to remove excess permeabilization buffer. Phalloidin-FITC conjugate solution in PBS was added to the cells at a concentration of 50 µg/mL and incubated for 30–60 min at room temperature, protected from light. Cells were washed twice with PBS to remove unbound phalloidin-FITC. Coverslips were mounted on microscope slides using a mounting medium containing DAPI. Stained cells were photographed using a confocal microscope. Images of the phalloidin-FITC fluorescence were collected and actin filament structures and their organization within the cells were visualized. The results were analyzed with the ImageJ 1.5.3 software for the mean fluorescence intensity (MFI), which indicates actin distribution in the cells. The values are displayed relative to those obtained in DMSO-treated vehicle control and the quantitative plots were generated using GraphPad Prism 5 software.

### 2.11. Assessing the Effect of MBZ on Surface Stem Cell Markers

The experiment was performed according to the standard protocol [28] with little modification. Briefly, OVCAR3 and OAW42 cell lines were cultured and treated with different concentrations of MBZ as described previously. After 48 h, the cells were harvested and washed with PBS. The cells were then resuspended in 50 µL of flow cytometry buffer. The required volume of PerCP Cy5.5-conjugated CD44 (CAT-560531, BD Biosciences, San Jose, CA, USA), FITC-conjugated CD24 (CAT-555427, BD Biosciences San Jose, CA, USA), and APC-conjugated EpCAM (CAT-555427, BD Biosciences, San Jose, CA, USA) was added according to the manufacturer’s description. The cells were then incubated at room temperature for 15 min in the dark. After 15 min, different treatment groups were acquired using BD FACSLyric^TM^ (BD Biosciences, San Jose, CA, USA) and BD FACSuite (version 1.4) software. Cell expression of all the stem cell markers was represented as a percentage of the total cell number. Each experiment was repeated at least 3 times. The change in expression of stem cell markers in different treatment groups was finally analyzed with FlowJo 10 software.

### 2.12. Analysis of MMP-9 Activity Using Gelatin Zymography

The experiment was performed following the standard protocol [29,30], with minor modifications. Briefly, the conditioned medium was collected and subjected to electrophoresis on a 10% SDS-PAGE gel containing 1.2 mg/mL gelatin under non-reducing conditions. The gels were then washed twice in 2.5% Triton X-100 and subsequently incubated in a calcium assay buffer. Following staining with Coomassie blue and destaining, gelatinolytic activity was visualized as negative staining bands. The zymographic bands were quantified using densitometry with ImageJ software and the quantitative plots were generated using GraphPad Prism 5 software. Each experiment was performed in triplicate to ensure consistency.

### 2.13. Cell Cycle Analysis by Flow Cytometry

PI-based cell cycle analysis by flow cytometry was performed to assess the role of MBZ on cell cycle arrest using standard protocol [31], incorporating minor adjustments. Subsequently, the cells were trypsinized, centrifuged at 2000 rpm for 7 min, washed with PBS, and fixed by adding 70% ethanol drop by drop while vortexing and incubating at 4 °C overnight. The fixed cell suspension was then centrifuged at 3000 rpm for 6 min and resuspended in PBS for washing. After centrifugation and discarding the supernatant, 1 mg/mL RNase A solution was added to the pellet and incubated at 37 °C for 3–4 h to eliminate all the RNA present in the cell. Following this, PI solution (50 µg/mL of PI) was added to the cells to bind only to the DNA just 15 min before analysis by flow cytometry. The fluorescence intensity of each group was immediately measured using BD FACSLyric^TM^ (BD Biosciences, USA) and flow cytometer, and cell count vs. PI-A histograms were generated on BD FACSuite (version 1.4) software following proper gating strategy to analyze the cell cycle phases. The results obtained were further processed and analyzed with FlowJo 10 software, and the quantitative plots were generated using GraphPad Prism 5. To confirm the results, the experiments were replicated at least three times.

### 2.14. Determining Mitochondrial Membrane Potential (ΔΨm) Using Flow Cytometry

Both the OC cell lines were cultured and administered with different concentrations of MBZ as described in previous methods. The protocol referenced in [32] was used to conduct the experiment, with a few modifications. Briefly, cells were detached from the culture flask using trypsin-EDTA, neutralized with culture media, and collected by centrifugation. Cells were washed twice with PBS and then Rhodamine123 was added to the cells at a recommended concentration of 5 μM and incubated for 30 min at 37 °C, protected from light. Cells were washed twice with PBS to remove unbound rhodamine123 stain. Cells were resuspended in a flow cytometry buffer and acquired using BD FACSLyric^TM^ (BD Biosciences, USA) and BD FACSuite (version 1.4) software. The change in fluorescence intensity of rhodamine123, which corresponds to the change in mitochondrial membrane potential (ΔΨm) of different treatment groups, was collected. The mean fluorescence intensity (MFI) of different treatment groups was finally analyzed with FlowJo 10 software. The values were compared to the values obtained in DMSO-treated vehicle control and the quantitative plots were generated using GraphPad Prism 5 software.

### 2.15. Detection of Cell Apoptosis by Flow Cytometry

Cells were cultured and treated with MBZ as previously outlined. Subsequently, the cells were harvested, washed, and stained using Annexin V/FITC and propidium iodide (PI) according to the manufacturer’s instructions (BioLegend). Finally, the stained cells were analyzed using BD FACSLyric^TM^ (BD Biosciences, USA) with fluorescence emission at 530 nm for Annexin V/FITC and 617 nm for PI. In the Annexin V/FITC vs. PI plot, cells positive for only Annexin V/FITC stain were considered to be at the early apoptotic phase while cells positive for both Annexin V/FITC and PI were considered to be at the late apoptotic phase. Cells positive for only PI stains were considered as necrotic population and the population of cells showing no positive stains were considered as live cells. All the phases were represented as a percentage of the total cell number. The results obtained were further processed and analyzed with FlowJo 10 software, and the quantitative plots were generated using GraphPad Prism 5. Each experiment was repeated at least 3 times.

### 2.16. p-NF-κB Translocation Analysis by Confocal Microscopy

We performed the experiment according to the protocol discussed in [33,34], incorporating some small adjustments. Briefly, OVCAR3 and OAW42 cells were seeded onto glass coverslips and treated with MBZ as previously described. Paraformaldehyde was added to the cell at a final concentration of 4% and incubated for 15–30 min at room temperature. Permeabilization buffer (0.1% Triton X-100) was added to the fixed cells and incubated for 10–15 min at room temperature. Cells were washed twice with PBS to remove excess fixative and permeabilization buffer. Nonspecific binding sites were blocked by incubating cells with a blocking buffer (1% BSA in PBST) for 30 min at room temperature. Cells were incubated with the primary antibody against p-NF-κB at a recommended concentration overnight at 4 °C. Cells were washed three times with PBS, followed by incubation with Alexa 488-tagged secondary antibody for 1 h at room temperature, protected from light. Cells were washed three times with PBS. Then, the coverslips were mounted on microscope slides using a mounting medium containing DAPI. Stained cells were photographed using a confocal microscope. Images of the fluorescent signal were collected to visualize p-NF-κB translocation and total p-Nf-κB (p65) (nucleus plus cytoplasm) expression. ImageJ 1.5.3 analysis software was used to quantify the nuclear localization of p-NF-κB by determining the nuclear-to-cytoplasmic fluorescence intensity ratios, and the quantitative plots were generated using GraphPad Prism 5 software.

### 2.17. Analysis of Protein Expression Using Immunoblotting

Both the OC cell lines were cultured and administered with different concentrations of MBZ as described in previous methods. We performed the experiment according to the standard procedure [31,35], with slight changes. Briefly, the cells in each well were then treated with 600 µL of RIPA lysis buffer solution, lysed on ice for 20 min, scraped with a cell scraper, and transferred into an Eppendorf tube. Total protein was obtained by collecting the supernatant after centrifugation. The Bradford method was used to determine the protein concentration of the extracted samples, and the sample volume was adjusted before electrophoresis. A total of 50 µg of samples were loaded into each well along with markers, and 10% Tris-Glycine gels were used to separate the protein samples under a constant current. The separated proteins were transferred to PVDF membranes. A solution of 5% BSA in TBST (TBS with 0.1% Tween-20) was used to block the membranes for 1 h at room temperature. Subsequently, the membranes were incubated overnight at 4 °C with specific primary antibodies targeting E-cadherin, N-cadherin, Vimentin, Cyclin B1, Cdc25C, Wee1, MMP-9, Bax, Bcl-2, Cleaved-PARP, Cleaved-Caspase-3, Cleaved-Caspase-9, Cleaved-Caspase-7, Cytochrome C, Girdin, p-Akt, p-IKKa/b, p-NF-kB (p65), and GAPDH. The next day, the membranes were washed with TBST three times and then incubated with the corresponding secondary antibody (CST, USA) for 1 h at room temperature in the dark. The membranes were scanned using the ChemiDoc Imaging System (Bio-Rad, Hercules, CA, USA). Finally, the relative gray values of the target proteins and internal reference proteins (GAPDH) were measured using ImageJ 1.5.3 Software, and the quantitative plots were generated using GraphPad Prism 5 software. Each experiment was performed in triplicate to ensure consistency.

### 2.18. Statistical Analysis

The results were presented as the mean ± standard error mean and statistical significance of data were analyzed using a one-way Anova test followed by Tukey’s test or a two-way Anova test followed by Bonferroni post-test or *t*-test using the GraphPad Prism software (version 5.0) (San Diego, CA, USA) and the following *p* values were considered significantly different: * *p* < 0.05; ** *p* < 0.01; *** *p* < 0.001.

## 3. Results

### 3.1. Mebendazole Eloquently Inhibits Cell Proliferation and Suppresses the In Vitro Tumorigenesis and Metastatic Parameters of OVCAR3 and OAW42 Cells

**Figure 1:**
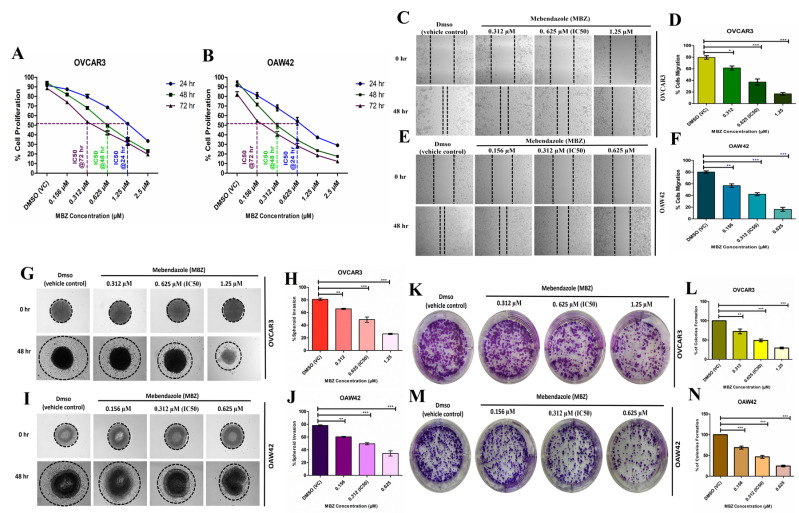
Mebendazole (MBZ) eloquently obstructs the cell proliferation of OVCAR3 and OAW42 cells in a dose- and time-dependent manner. (**A**,**B**) Cells were treated with MBZ at varying concentrations (0.156, 0.312, 0.625, 1.25, and 2.5 µM) and DMSO vehicle control (VC) for different time points (24, 48, and 72 h). Cell proliferation was assessed using the MTT assay. The half-maximal inhibitory concentration (IC_50_) of MBZ was determined for each time point, and a quantitative plot was generated using GraphPad Prism software. Data represent the mean ± standard error of the mean of two independent experiments in triplicate. Additionally, Mebendazole (MBZ) significantly impedes cell migration, spheroid invasion, and colony formation in both cell lines. (**C**,**E**,**G**,**I**,**K**,**M**) Represent microscopic images of cell migration (magnification 10×, scale bar = 100 μm), spheroid invasion (magnification 10×, scale bar = 100 μm), and colony formation assay (digital camera, 2× Zoom) after 48 h of treatment at the indicated concentration of MBZ studied. Further, (**D**,**F**,**H**,**J**,**L**,**N**) represent the quantitative plots of percentage (%) values of areas of cell migration, spheroid invasion, and number of colonies formation. The bars represent the mean ± standard error of the mean of three independent experiments. Statistical significance was determined by one-way ANOVA, with * *p* < 0.05, ** *p* < 0.01, and *** *p* < 0.001 considered statistically significant and ns: not significantcompared to the DMSO-treated vehicle control (VC).

To assess the effect of MBZ on cell proliferation, OVCAR3 and OAW42 ovarian cancer (OC) cell lines were treated with varying concentrations of MBZ (0.156, 0.312, 0.625, 1.25, and 2.5 µM) for different time points (24, 48, and 72 h). Cell proliferation was assessed using the MTT assay. Our results revealed that MBZ significantly inhibited cell proliferation in both cell lines in a dose-dependent and time-dependent manner. The inhibitory effect of MBZ was more pronounced at higher concentrations and longer incubation times. Further analysis revealed that 48 h was the optimal time point for MBZ treatment as the cell density increased from 24 to 48 h but decreased from 48 h to 72 h in the DMSO vehicle control (VC) wells, suggesting that 48 h represents a balance between cell growth and MBZ-induced inhibition (Figure 1A,B). The IC_50_ (concentrations required to inhibit cell proliferation by 50%) values for MBZ were determined in OVCAR3 and OAW42 cells at different time points (Table 1).

These findings indicate that OAW42 cells were more sensitive to MBZ-induced inhibition than OVCAR3 cells, as evidenced by the lower IC_50_ values for OAW42. The observed dose-dependent and time-dependent inhibition of cell proliferation, coupled with the determination of IC_50_ values, strongly suggest that MBZ can effectively target and suppress the growth of ovarian cancer (OC) cells. Furthermore, as 48 h was the optimal time point, IC_25_, IC_50_, and IC_75_ values of MBZ were selected for the respective cell lines to establish the dose-dependent response of MBZ for further functional experiments (Table 2).

Similar to MBZ, Cisplatin exhibits a dose-dependent inhibitory effect on both cell lines. However, MBZ demonstrates lower IC_50_ values (0.625 μM for OVCAR3 and 0.312 μM for OAW42) compared to Cisplatin (2.5 μM for OVCAR3 and 1.25 μM for OAW42) (Supplementary Figure S1). This indicates that MBZ requires significantly lower concentrations to achieve 50% cell growth inhibition compared to Cisplatin. Furthermore, OAW42 cells exhibit higher sensitivity to both drugs compared to OVCAR3, as evidenced by the steeper slope of the OAW42 curves and the lower IC_50_ values. These results suggest that MBZ may have a higher potency than Cisplatin in these ovarian cancer cell lines, warranting further investigation into its potential as a promising therapeutic agent.

We also investigated the effects of MBZ on cell migration; a scratch wound assay was performed on OVCAR3 (Figure 1C,D) and OAW42 (Figure 1E,F) cell lines. This assay involves creating a wound in a confluent monolayer of cells and measuring the rate at which cells migrate to close the wound. MBZ showed an apparent dose-dependent inhibition of cell migration. As the concentration of MBZ increased, the area of cell migration decreased. Exposure to MBZ concentrations of 0.312, 0.625 (IC_50_), and 1.25 µM reduced the migration area of OVCAR3 cells to 61.5% (*p* < 0.05), 37% (*p* < 0.001), and 17% (*p* < 0.001), respectively, from 80% in DMSO (VC). In OAW42 cells, MBZ concentrations of 0.156, 0.312 (IC_50_), and 0.625 µM reduced the migration area to 57% (*p* < 0.01), 42% (*p* < 0.001), and 16.4% (*p* < 0.001), respectively, from 80% in DMSO (VC).

Likewise, we analyzed the invasive potential of OC spheroids originating from the OVCAR3 and OAW42 cell lines. The treatment with MBZ markedly inhibited the invasive regions of both OVCAR3 (Figure 1G,H) and OAW42 (Figure 1I,J) spheroids in a dose-dependent manner. In relation to the vehicle control (DMSO), MBZ concentrations of 0.312, 0.625 (IC_50_), and 1.25 µM decreased the invasion area of OVCAR3 cells to 66% (*p* < 0.01), 48.8% (*p* < 0.001), and 26% (*p* < 0.001), respectively, from 80% in DMSO (VC). In contrast, for OAW42 cells, MBZ concentrations of 0.156, 0.312 (IC_50_), and 0.625 µM similarly reduced the spheroid invasion area to 60.3% (*p* < 0.01), 49.5% (*p* < 0.001), and 34.3% (*p* < 0.001), respectively, from 78% in DMSO (VC).

We also examined the effect of MBZ on the clonogenic potential of OVCAR3 (Figure 1K,L) and OAW42 (Figure 1M,N) cells. Following treatment of MBZ at the indicated doses for 48 h, the cells were changed to complete fresh media and incubated without the drugs for an additional 10 days. MBZ treatment typically reduced the number of colonies formed in a dose-dependent manner, indicating the inhibition of OC cell proliferation and survival. Relative to the vehicle control (DMSO), the administration of MBZ at concentrations of 0.312, 0.625 (IC_50_), and 1.25 µM resulted in a decrease in the number of colonies of OVCAR3 cells to 72.3% (*p* < 0.01), 49% (*p* < 0.001), and 29.3% (*p* < 0.001), respectively. Conversely, the OAW42 cell line exhibited a comparable reduction in the number of colonies of 69% (*p* < 0.001), 46.7% (*p* < 0.001), and 24.7% (*p* < 0.001) with MBZ concentrations of 0.156, 0.312 (IC_50_), and 0.625 µM, respectively.

### 3.2. MBZ Disrupts the Cytoskeleton Network by Impeding Actin Polymerization

**Figure 2:**
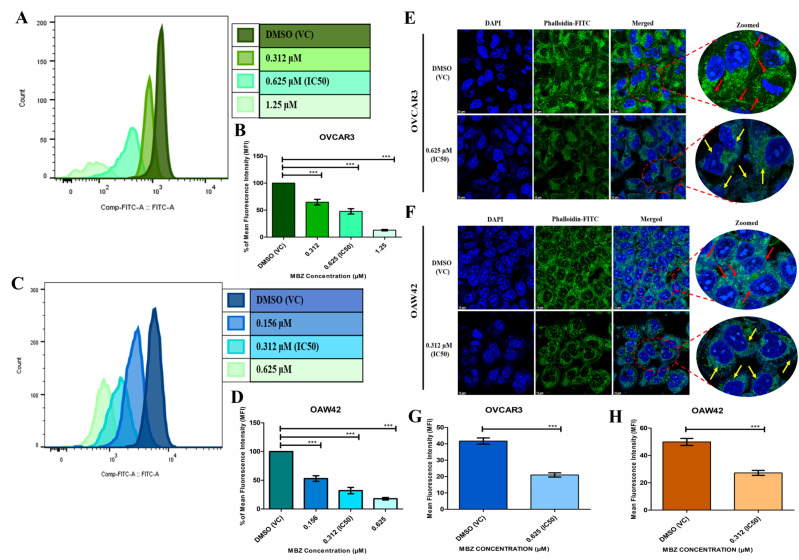
MBZ significantly hinders the actin polymerization in OC cell lines in a dose-dependent manner. OVCAR3 and OAW42 cells were treated with different concentrations of MBZ and DMSO vehicle control (VC) for 48 h and were determined by staining the cells with Phalloidin-FITC and analyzed using a flow cytometer. (**A**,**C**) signify the histogram overlays shift plot and (**B**,**D**) show a graphical representation of the quantitative change in the percentage of mean fluorescence intensity (MFI) shifts, which indicate waning in actin polymerization. (**E**,**F**) denote the confocal microscopic images of Phalloidin-FITC staining of actin filaments of OVCAR3 and OAW42 cells and counter staining with DAPI. The red arrow in the zoomed image of DMSO (vehicle control (VC)) indicates the intact actin filamentous network of individual cells with organized cytoskeleton while the yellow arrow shows the disrupted actin filamentous network with disordered cytoskeleton arrangement in MBZ (IC_50_)-treated OVCAR3 and OAW42 cells (scale bar = 10 µm). (**G**,**H**) indicate the quantitative plots of the percentage of mean fluorescence intensity (MFI) shifts, which signify decrease in actin polymerization in both the cell lines compared to DMSO (vehicle control (VC)). The bars represent the mean ± standard error of the mean of three independent experiments. Level of significance (*: *p* < 0.05, **: *p* < 0.01, ***: *p* < 0.001, and ns: not significant) compared to DMSO (vehicle control (VC)), performed using 1-way ANOVA test or *t*-test.

We conducted flow cytometry analysis to understand the role of actin polymerization in ovarian cancer cell metastasis. The histograms displayed the staining profile of FITC-phalloidin-stained intracellular actin, corresponding to the amount of F-actin in OVCAR3 and OAW42 cells, which was measured and analyzed via flow cytometry (Figure 2). Treatment with MBZ significantly inhibited actin polymerization in a dose-dependent manner, evident from the notable decrease in FITC-phalloidin mean fluorescence intensity (MFI) shifts in the histogram overlays (Figure 2A,C). To mitigate the effects of fluorescence intensity variation across different MBZ doses, the change in MFI data was normalized to the change in the percentage and compared with the vehicle control (DMSO) to analyze differences in actin polymerization. In OVCAR3 cells, the MFI percentages were 65% (*p* < 0.001), 48% (*p* < 0.001), and 13% (*p* < 0.001) at MBZ concentrations of 0.312, 0.625 (IC_50_), and 1.25 µM, respectively, (Figure 2B). Conversely, in OAW42 cells, the MFI percentages were 53% (*p* < 0.001), 32% (*p* < 0.001), and 18% (*p* < 0.001) at MBZ concentrations of 0.156, 0.312 (IC_50_), and 0.625 µM, respectively, (Figure 2D).

We also analyzed the effects of MBZ on cell integrity by examining actin polymerization in OVCAR3 and OAW42 cells using FITC-phalloidin staining observed under confocal microscopy. Cells treated with MBZ at their respective IC_50_ concentrations (0.625 µM for OVCAR3 and 0.312 µM for OAW42) for 48 h exhibited a disorganized cytoskeletal arrangement in both the cell lines. In contrast, DMSO (VC) cells displayed an intact actin filament network with a dense and organized cytoskeleton in both OVCAR3 (Figure 2E) and OAW42 (Figure 2F). Additionally, MFI shifts were quantified using ImageJ. The quantification plot revealed a substantial decline in MFI, indicating a marked reduction in actin distribution, which also validated the result of flow cytometry analysis. In OVCAR3 cells, MFI decreased to 50% (*p* < 0.001) at 0.625 µM MBZ compared to DMSO (VC), while in OAW42 cells, MFI was reduced to 54% (*p* < 0.001) at 0.312 µM MBZ relative to DMSO (VC).

### 3.3. MBZ Substantially Down-Regulates Stem Cell Markers, Gelatinolytic Activity and Modulates the Metastasis-Related Proteins

**Figure 3:**
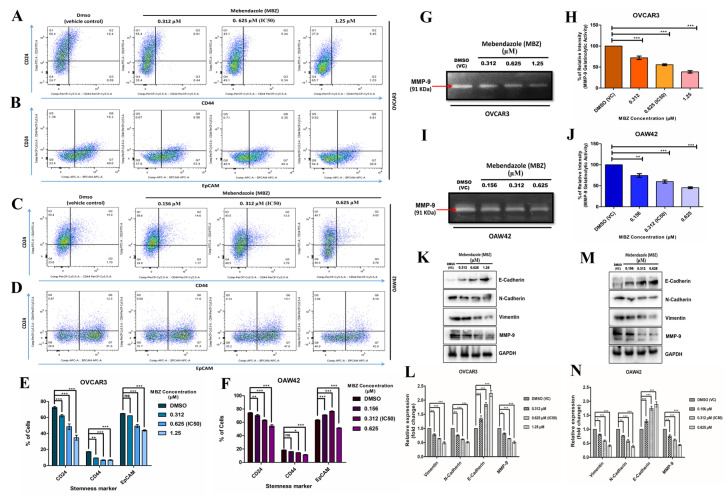
(**A**–**F**) Mebendazole (MBZ) ardently disrupts the cancer stemness characteristics of both OVCAR3 and OAW42 cells in a dose-dependent manner, analyzed by flow cytometry. (**A**) CD24 vs. CD44 and (**B**) CD44 vs. EPCAM dot plot showing a decrease in the percentage of CD24-, CD44-, and EPCAM-expressing cells with increasing concentration of mebendazole in the OVCAR3 cell line. In the OAW42 cell line, (**C**) CD24 vs. CD44 dot plot showed a similar pattern of decrease in % of expression as OVCAR3 cells. However, (**D**) CD44 vs. EPCAM dot plot of OAW42 cell line shows no dose-dependent decrease pattern in % of EPCAM-expressing cells. (**E**,**F**) show a graphical representation of change in CD24, CD44, and EPCAM expression with increasing concentration of Mebendazole in OVCAR and OAW42 cell lines, respectively. (**G**–**N**) MBZ also thwarts gelatinolytic activity and modulates the metastasis-related proteins in OC cell lines in a dose-responsive way. (**G**,**I**) represent the gelatin zymography of MMP-9 activity alterations in OVCAR3 and OAW42 cells, and supernatant was collected after treatment with different concentrations of MBZ and DMSO vehicle control for 48 h. (**H**,**J**) The gelatinolytic activity of MMP-9 at different MBZ concentrations was calculated and graphed using GraphPad Prism software. (**K**,**M**) The expression of proteins involved in MBZ-dissuaded metastasis potential was analyzed by Western blot, with GAPDH as the loading control. (**L**,**N**) represent the quantitative graphs of the expression level of proteins in fold change. Western blot and quantitative analysis revealed that as the MBZ concentration increases, the expression of N-cadherin, vimentin, and MMP-9 reduces while the expression of E-cadherin increases significantly compared to DMSO vehicle control (VC). The bars represent the mean ± standard error mean of three independent experiments. Level of significance (*: *p* < 0.05, **: *p* < 0.01, ***: *p* < 0.001, and ns: not significant) compared to DMSO (vehicle control (VC)), performed using 1-way or 2-way ANOVA test.

The cancer stem cell (CSC) property of OC is a crucial contributor to metastasis and drug resistance. Thus, we checked the effect of MBZ on the expression of surface stem cell markers. Like other experiments, the OVCAR3 and OAW42 cells were treated with 0.312, 0.625 (IC_50_), and 1.25 µM for OVCAR3 and 0.156, 0.312 (IC_50_), and 0.625 µM for OAW42, respectively. Flowcytometric analysis showed MBZ treatment decreased the CD24, CD44, and EpCAM in OVCAR3 and OAW42 cell lines compared to the only DMSO-treated group. However, in the OVCAR3 cell line, the MBZ treatment groups showed more significant changes in surface marker expressions than the OAW42 cell line, especially CD24 and CD44 levels. In the OVCAR3 cell line, exposure to MBZ concentrations at 0.312, 0.625 (IC_50_), and 1.25 µM showed a significant decrease in the level of CD24 to 62.1% (*p* < 0.001), 48.5% (*p* < 0.001), and 34.7% (*p* < 0.001), respectively, when compared with only DMSO (VC)-treated cells showing 72.4% of CD24-expressing cells. Likewise, CD44 expression was also bated to 13% (*p* < 0.01), 10.93% (*p* < 0.001), and 9.72% (*p* < 0.001), respectively, from 19.94% in DMSO (VC), and the expression of EpCAM was ablated to 63.83% (ns), 52.93% (*p* < 0.001), and 47.5% (*p* < 0.001), respectively, from 70.47% in DMSO (VC) (Figure 3A,B,E). On the other hand, in OAW42 cells, the change in CD24 and CD44 expression was observed in all the concentrations. CD24 expression was decreased to 70.27% (*p* < 0.01) in 0.156 µM, 63.1% (*p* < 0.001) in 0.312 µM, and 54.4% (*p* < 0.001) in 0.625 µM compared to 74.2% in DMSO (VC). The CD44 level also reduced to 16.1% (ns), 14.4% (*p* < 0.05), and finally 11.3% (*p* < 0.001), respectively, compared to 18.2% in DMSO (VC). Nonetheless, the change in expression of EpCAM was not concentration-dependent; instead, it was found to be increased in 0.156 µM (71.03%—*p* < 0.001) and 0.312 µM (76.47%—*p* < 0.001) MBZ concentration but decreased in 0.625 µM (51.47%—(*p* < 0.001) when compared with the DMSO (VC) group (63.43%) (Figure 3C,D,F).

Furthermore, we performed gelatin zymography to determine the functional role of MBZ in regulating MMP-9 activity. Our results revealed that MBZ significantly inhibited MMP-9 activity in all tested concentrations of MBZ after 48 h of treatment compared to DMSO (VC) in both cell lines. The percentage of MMP-9 gelatinolytic activity in OVCAR3 cells was noted as 72% (*p* < 0.001), 55.5% (*p* < 0.001), and 38.5% (*p* < 0.001) for MBZ concentrations of 0.312, 0.625 (IC_50_), and 1.25 µM, respectively, (Figure 3G,H). On the contrary, OAW42 cells demonstrated MMP-9 gelatinolytic activity of 74.5% (*p* < 0.01), 60% (*p* < 0.001), and 45% (*p* < 0.001) at MBZ concentrations of 0.156, 0.312 (IC_50_), and 0.625 µM (Figure 3I,J). These findings revealed that MBZ markedly inhibited the secretion of MMP-9 in a concentration-dependent manner.

Our results also showed that exposure to MBZ (0.312 µM, 0.625 µM, and 1.25 µM) significantly reduced the levels of metastasis-associated proteins in OVCAR3 cells, with MMP-9 expression at 0.82-fold (*p* < 0.05), 0.64-fold (*p* < 0.001), and 0.51-fold (*p* < 0.001); N-Cadherin at 0.75-fold (*p* < 0.001), 0.65-fold (*p* < 0.001), and 0.51-fold (*p* < 0.001); and Vimentin at 0.78-fold (*p* < 0.01), 0.64-fold (*p* < 0.001), and 0.5-fold (*p* < 0.001), respectively. Conversely, E-Cadherin levels increased to 1.35-fold (*p* < 0.01), 1.85-fold (*p* < 0.001), and 2.24-fold (*p* < 0.001) at the same concentrations compared to the DMSO (VC) (Figure 3K,L). Treatment of OAW42 cells with MBZ (0.156 µM, 0.312 µM, and 0.625 µM) showed similar trends, with MMP-9 at 0.76-fold (*p* < 0.01), 0.61-fold (*p* < 0.001), and 0.44-fold (*p* < 0.001); N-Cadherin at 0.77-fold (*p* < 0.01), 0.58-fold (*p* < 0.001), and 0.39-fold (*p* < 0.001); Vimentin at 0.81-fold (*p* < 0.05), 0.58-fold (*p* < 0.001), and 0.41-fold (*p* < 0.001); and E-Cadherin at 1.3-fold (*p* < 0.001), 1.75-fold (*p* < 0.001), and 1.9-fold (*p* < 0.001) (Figure 3M,N). These findings indicate that MBZ disrupts the metastatic machinery by down-regulating key proteins in a dose-dependent manner, potentially explaining its ability to hinder metastasis.

### 3.4. Mebendazole Substantially Induces G2/M Cell Cycle Arrest

**Figure 4:**
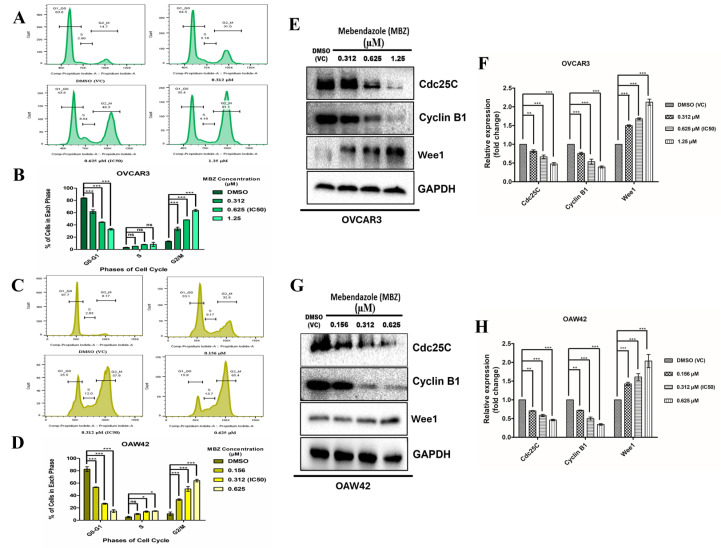
Mebendazole induces G2/M cell cycle arrest in ovarian cancer cell lines dose-dependently. (**A**,**C**) show the histogram plots of cell cycle alterations in OVCAR3 and OAW42 cells treated with indicated concentrations of the MBZ and DMSO vehicle control for 48 h. (**B**,**D**) indicate the quantitative plots of the percentage of cells in each phase of the cell cycle at different MBZ concentrations. (**E**,**G**) The expression level of proteins involved in MBZ-mediated cell cycle arrest was analyzed by Western blot, with GAPDH as the loading control. (**F**,**H**) denote the quantitative plots of expression of proteins in fold change. Western blot and quantitative analysis revealed that as the MBZ concentration increases it reduces the expression of Cdc25C, and cyclin B1 while increasing the expression of Wee1 substantially compared to the DMSO vehicle control (VC). The bars represent the mean ± standard error mean of three independent experiments in triplicate. Level of significance (*: *p* < 0.05, **: *p* < 0.01, ***: *p* < 0.001, and ns: not significant) compared to DMSO (vehicle control (VC)), performed using 1-way or 2-way ANOVA test.

MBZ has been previously reported to induce G2/M cell cycle arrest in various cancers [35]. To validate the impact of MBZ exposure on the cell cycle of OC cell lines, we treated both OC cell lines with MBZ at concentrations of 0.312, 0.625 (IC_50_), and 1.25 µM for OVCAR3 and 0.156, 0.312 (IC_50_), and 0.625 µM for OAW42, along with DMSO (VC) for 48 h, and conducted the PI-based cell cycle analysis using flowcytometry. We observed that MBZ treatment resulted in a G2/M block and eventually a gradual increase in the DNA content of the G2/M phase with an increasing concentration of MBZ. Subsequently, a noticeable reduction in DNA content was also observed in the G0/G1 phase with an increasing concentration of MBZ when compared with the DMSO (VC)-treated group in both cell lines. Briefly, in DMSO (VC), a traditional histogram pattern of the cell cycle was observed in both cell lines. In OVCAR3 cells, a high G0/G1 peak of 84%, with the lowest population of 4% in the S phase, and a small peak of 13% cell population in the G2/M phase were observed. Similarly, in OAW42 cells, 82% of the population in the G0/G1 phase, 5% in the S phase, and 11% in the G2/M phase were observed. In the OVCAR3 cell line, upon MBZ treatment at 0.312 µM concentration, the population at the G0/G1 phase was reduced to 62% (*p* < 0.001), and not much change in S phase was observed with 5% (ns) cell population, and the G2/M phase was increased to 33% (*p* < 0.001). Correspondingly at higher concentrations, the G0/G1 phase showed a gradual decrease, i.e., 45% (*p* < 0.001) for 0.625 µM (IC_50_) and 33% (*p* < 0.001) for 1.25 µM. The S phase also mildly increased: 8% (ns) for 0.625 µM (IC_50_) and 8.5% (ns) for 1.25 µM. The G2/M phase was gradually increased, i.e., 48% (*p* < 0.001) for 0.625 µM (IC_50_) and 65% (*p* < 0.001) for 1.25 µM, (Figure 4A,B). Similarly, in the OAW42 cell line, MBZ treatment at 0.156 µM resulted in 54% (*p* < 0.001) cell population in the G0/G1 phase, 10% (ns) in the S phase, and 33% (*p* < 0.001) in the G2/M phase. At 0.312 µM (IC_50_) concentration, the cell population was reduced to 28% (*p* < 0.001) in the G0/G1, 14% (*p* < 0.05) in the S phase, and G2/M phase was increased to 51% (*p* < 0.001). At the highest concentration of 0.625 µM, the percentage was reduced to 16% (*p* < 0.001) in the G0/G1 phase. A mild increase in the S phase, i.e., 15% (*p* < 0.05), was observed, and the highest cell population of 64% (*p* < 0.001) was observed in the G2/M phase (Figure 4C,D).

To investigate the mechanism of MBZ-induced cell cycle arrest, we conducted a Western blot analysis of Cdc25C, Cyclin B1, and Wee1 levels. Our results showed that MBZ significantly reduced G2/M phase-associated proteins in OVCAR3 cells, with Cdc25C levels recorded at 0.81-fold (*p* < 0.01) at 0.312 µM, 0.66-fold (*p* < 0.001) at 0.625 µM, and 0.47-fold (*p* < 0.001) at 1.25 µM. Cyclin B1 levels were recorded at 0.75-fold (*p* < 0.001), 0.53-fold (*p* < 0.001), and 0.39-fold (*p* < 0.001) at the same concentrations. In contrast, Wee1 levels increased, measuring 1.5-fold (*p* < 0.001) at 0.312 µM, 1.68-fold (*p* < 0.001) at 0.625 µM, and 2.12-fold (*p* < 0.001) at 1.25 µM compared to the DMSO (VC) (Figure 4E,F). Similar trends were observed in OAW42 cells. Cdc25C expression was recorded at 0.70-fold (*p* < 0.01) at 0.156 µM, 0.58-fold (*p* < 0.001) at 0.312 µM, and 0.46-fold (*p* < 0.001) at 0.625 µM, while Cyclin B1 levels were recorded at 0.72-fold (*p* < 0.01), 0.5-fold (*p* < 0.001), and 0.34-fold (*p* < 0.001) at the same concentrations. Meanwhile, Wee1 expression in OAW42 cells was 1.42-fold (*p* < 0.001) at 0.156 µM, 1.6-fold (*p* < 0.001) at 0.312 µM, and 2-fold (*p* < 0.001) at 0.625 µM relative to the DMSO (VC) (Figure 4G,H). These findings indicate that MBZ selectively down-regulates G2/M-related proteins in both OVCAR3 and OAW42 cell lines in a concentration-dependent response, leading to cell cycle arrest at the G2/M phase.

### 3.5. Mebendazole Notably Disrupts Mitochondrial Membrane Potential (ΔΨm) and Induces Caspase-3-Dependent Intrinsic Pathway Program Cell Death (Apoptosis)

**Figure 5:**
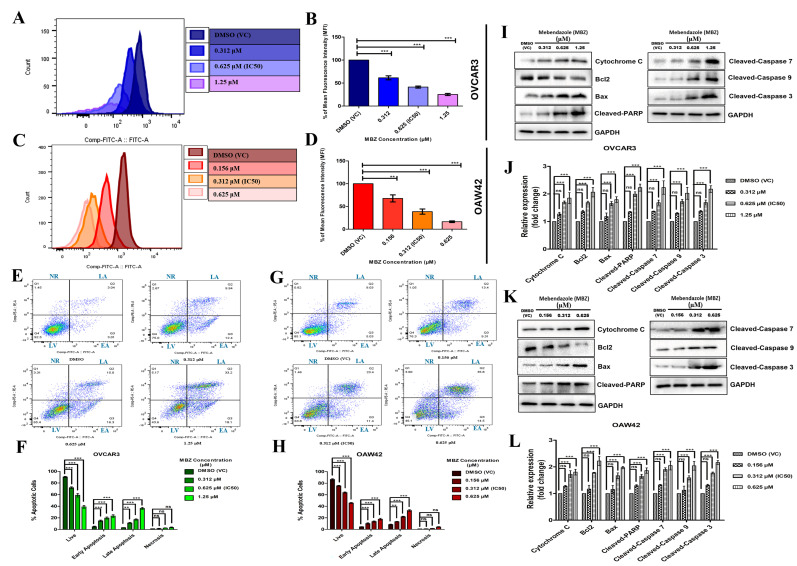
Mebendazole notably disrupts mitochondrial membrane potential and induces apoptosis in ovarian cancer cell lines in a dose-dependent manner. OVCAR3 and OAW42 cells were treated with different concentrations of MBZ and DMSO vehicle control for 48 h and were determined by staining the cells with Rhodamine123 and analyzed using a flow cytometer. (**A**,**C**) signify the histogram overlays shift plot and (**B**,**D**) shows a graphical representation of quantitative change in the percentage of mean fluorescence intensity (MFI) shifts, which indicates depolarization of mitochondrial membrane potential. (**E**,**G**) show the dot plot of apoptosis in OVCAR3 and OAW42 cells treated with different concentrations of the MBZ or DMSO vehicle control for 48 h and was analyzed by flow cytometry using FITC-Annexin V/PI. (**F**,**H**) indicate the quantitative plots of the percentage of cells of live (LV), early apoptosis (EA), late apoptosis (LA), and necrosis (NR). (**I**,**K**) represent the blot image of the expression of proteins involved in MBZ-induced apoptosis, analyzed using Western blot, with GAPDH as the loading control. (**J**,**L**) denote the quantitative plots of the level of expression of proteins in fold change. Western blot analysis showed that as the MBZ concentration increases, it increases the apoptotic protein expression (Cytochrome C, Bax, cleaved-PARP, cleaved-Caspase 3, cleaved-Caspase 7, and cleaved-Caspase 9) while reducing the expression of an anti-apoptotic protein (Bcl2) significantly compared to DMSO (vehicle) control. The bars represent the mean ± standard error of the mean of three independent experiments in triplicate. Level of significance (*: *p* < 0.05, **: *p* < 0.01, ***: *p* < 0.001, and ns: not significant) compared to DMSO (vehicle control (VC)), performed using 1-way or 2-way ANOVA test. LV: live; EA: early apoptosis; LA: late apoptosis; and NR: necrosis.

We performed flow cytometry to analyze the mitochondrial membrane potential (ΔΨm), essential for cellular energy metabolism and programmed cell death. The histograms represented the Rhodamine123 fluorescence intensity, reflecting the active mitochondrial membrane potential of OVCAR3 and OAW42 cells. A dose-dependent decrease in ΔΨm was observed following MBZ treatment, as reflected by the significant shift in rhodamine123 fluorescence intensity depicted in the histogram overlays (Figure 5A,C). To account for variations in fluorescence intensity at different MBZ doses, the changes in MFI data were normalized to percentage shifts in mean fluorescence intensity (MFI) and compared with the vehicle control (DMSO). In OVCAR3 cells, MFI percentages were recorded at 62% (*p* < 0.001), 41.5% (*p* < 0.001), and 25.2% (*p* < 0.001) for MBZ concentrations of 0.312, 0.625 (IC_50_), and 1.25 µM, respectively, (Figure 5B). In contrast, OAW42 cells showed MFI percentages of 68% (*p* < 0.01), 39% (*p* < 0.001), and 16.5% (*p* < 0.001) at 0.156, 0.312 (IC_50_), and 0.625 µM of MBZ (Figure 5D). These observations imply that MBZ potentially affects mitochondrial membrane potential dysfunction and energy dynamics in OVCAR3 and OAW42 cells, which may further influence the apoptotic responses.

To further investigate the apoptosis-inducing property of MBZ on OC cells, dual staining by Annexin V and PI was used to determine the different phases of apoptosis. In the Annexin V vs. PI plot, only Annexin V-positive cells were characterized as the early apoptotic cells, and PI-positive cells were characterized as the necrotic cells. Annexin V- and PI-positive cells were characterized as late apoptotic cells. Our study, with an increasing concentration of MBZ, showed an increase in early and late apoptosis in both OVCAR3 and OAW42 cell lines, and gradatim decreased in a live cell population. Briefly, in the OVCAR3 cell line, the treatment with 0.312, 0.625 (IC_50_), and 1.25 µM concentration of MBZ showed an increase in early apoptotic population to 15% (*p* < 0.001), 19.8% (*p* < 0.001), and 23.2% (*p* < 0.001), respectively, from only 4.9% in the DMSO (VC) group. The late apoptotic population also progressively increased to 11.3% (*p* < 0.01), 17.2% (*p* < 0.001), and 36.4% (*p* < 0.001) from 3.3% in the DMSO (VC) group. The necrotic population mildly increased with increasing concentration but was insignificant (Figure 5E,F). Similar trends were observed in OAW42 cells. With exposure to MBZ concentrations at 0.156, 0.312 (IC_50_), and 0.625 µM, the early apoptotic population showed a rise in gradatim to 10.2% (*p* < 0.001), 13.6% (*p* < 0.001), and 17.66% (*p* < 0.001), respectively, from only 4.5% in DMSO (VC) group. The late apoptotic population also progressively increased to 13.4% (*p* < 0.01), 21.9% (*p* < 0.001), and 32.7% (*p* < 0.001) from 8.3% in the DMSO (VC) group. Although a mild rise in necrotic cells was observed with increasing MBZ concentrations, this effect was not statistically significant (Figure 5G,H).

Our findings showed that MBZ notably attenuated the mitochondrial membrane potential and induced apoptosis dose-dependently. Therefore, we utilized Western blot analysis to elucidate the mechanism of MBZ-mediated apoptosis. Exposure to MBZ at 0.312, 0.625 (IC_50_), and 1.25 µM significantly increased pro-apoptotic proteins in OVCAR3 compared to DMSO (VC): Bax (1.17 (ns), 1.65 (*p* < 0.001), 1.8 (*p* < 0.001)-fold); cytochrome C (1.27 (ns), 1.7 (*p* < 0.001), 1.85 (*p* < 0.001)-fold); cleaved-PARP (1.35 (ns), 2 (*p* < 0.001), 2.3 (*p* < 0.001)-fold); cleaved caspase-3 (1.4 (ns), 1.7 (*p* < 0.001), 2.2 (*p* < 0.001)-fold); cleaved caspase-7 (1.36 (ns), 1.6 (*p* < 0.001), 2.2 (*p* < 0.001)-fold); and cleaved caspase-9 (1.3 (ns), 1.72 (*p* < 0.001), 2 (*p* < 0.001)-fold). Conversely, Bcl2 levels decreased 1.36 (ns), 1.68 (*p* < 0.001), and 2 (*p* < 0.001)-fold with respect to DMSO (VC) (Figure 5I,J). Similarly, in OAW42 cells upon MBZ exposure (0.156, 0.312 (IC_50_), and 0.625 µM), pro-apoptotic proteins were elevated: Bax 1.2 (ns), 1.8 (*p* < 0.001), 2.2 (*p* < 0.001)-fold); cytochrome c (1.27 (ns), 1.72 (*p* < 0.001), 1.8 (*p* < 0.001)-fold); cleaved-PARP (1.33 (ns), 1.65 (*p* < 0.001), 1.86 (*p* < 0.001)-fold); cleaved caspase-3 (1.3 (ns), 1.76 (*p* < 0.001), 2.2 (*p* < 0.001)-fold); cleaved caspase-7 (1.32 (ns), 1.9 (*p* < 0.001), 2 (*p* < 0.001)-fold); and cleaved caspase-9 (1.1 (ns), 1.6 (*p* < 0.001), 2 (*p* < 0.001)-fold), while Bcl2 decreased to 1.2 (ns), 1.8 (*p* < 0.001), and 2.2 (*p* < 0.001)-fold, respectively, in comparison to DMSO (VC) (Figure 5K,L). The results herein suggested that MBZ can elicit intrinsic apoptosis in both ovarian cancer cell lines, with the apoptotic response and the increasing concentration of MBZ.

### 3.6. MBZ Significantly Down-Regulates the Expression of p-Nf-κB and Its Translocation from Cytoplasm to the Nucleus and Exerts Its Anticancer Effects Through a Novel Girdin-Mediated Akt/IKKα/β/NF-κB Signaling Axis

**Figure 6:**
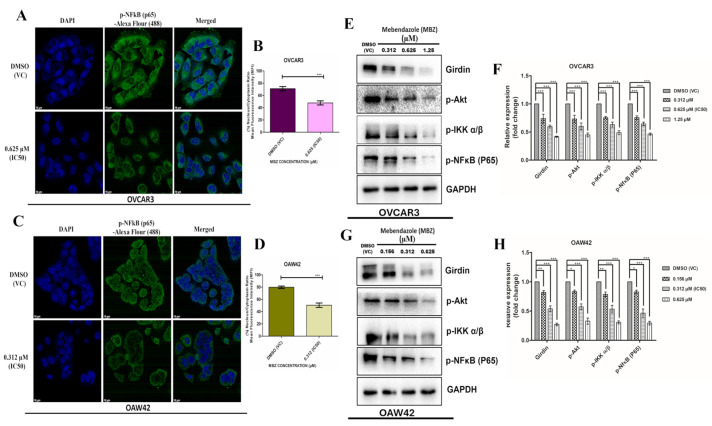
MBZ significantly fettered the expression of p-Nf-κB and its translocation from cytoplasm to the nucleus in both the cell lines after the treatment of MBZ (IC_50_) for 48 h and the images were acquired through a confocal microscope (scale bar = 10 µm). (**A**,**C**) denote the confocal microscopic images of OVCAR3 and OAW42 cells, after immunofluorescence (IF) staining with antibody against p-Nf-κB (p65) and Alexa 488-tagged secondary antibody and counter-stained with DAPI. (**B**,**D**) indicate the quantitative plots of the percentage of MFI of nucleus vs. cytoplasm ratio, which signifies a decrease in translocation of p-Nf-κB (p65) expression compared to DMSO (vehicle control (VC) in both the cell lines. MBZ substantially exerts its anticancer effects on OVCAR3 and OAW42 cells through a novel Girdin-mediated Akt/IKKα/β/NF-κB signaling axis. (**E**,**G**) show the blot image of Girdin, p-Akt, p-IKKα/β, and p-Nf-κB determined by Western blot with GAPDH as the loading control. (**F**,**H**) represent the quantitative plots of expression of proteins. Western blot and quantitative analysis revealed that as the MBZ concentration increases, it reduces the expression of Girdin, p-Akt, p-IKKα/β, and p-Nf-κB substantially compared to DMSO (vehicle control (VC)) in both the cell lines. The bars represent the mean ± standard error of the mean of three independent experiments. Level of significance (*: *p* < 0.05, **: *p* < 0.01, ***: *p* < 0.001, and ns: not significant) compared to DMSO (vehicle control (VC)), performed using a 2-way ANOVA test or *t*-test.

The cells were exposed to MBZ with IC_50_ concentrations (0.625 µM for OVCAR3 and 0.312 µM for OAW42) for 48 h and stained with antibody against p-Nf-κB (p65) and Alexa 488-tagged secondary antibody and counter-stained with DAPI (Figure 6A,C). The photomicroscopic images and the quantitative plot indicate that MBZ-treated cells showed a significant decline in the percentage of MFI of the nucleus vs. cytoplasm ratio, which indicates the decreased in the translocation of p65 from cytoplasm to the nucleus in comparison to DMSO (VC) (Figure 6B,D).

Previous studies have shown that Girdin modulates various cancer properties including cell proliferation, metastasis, cytoskeleton structure, and apoptosis [4]. Therefore, to shed light on the effect of MBZ on Girdin and the signaling axis through which it shows the anticancer effect in OVCAR3 and OAW42 cells, we conducted a Western blot analysis to assess the levels of Girdin, p-Akt, p-IKKα/β, and p-NF-κB. Our results revealed that exposure to MBZ at 0.312, 0.625 (IC_50_), and 1.25 µM in OVCAR3 cells led to a significant dose-response decrease in the levels of Girdin by 0.74-fold (*p* < 0.001), 0.6-fold (*p* < 0.001), and 0.41 (*p* < 0.001)-fold respectively. Similarly, at the same MBZ concentrations, p-Akt levels decreased by 0.73 (*p* < 0.001)-fold, 0.6 (*p* < 0.001)-fold, and 0.45 (*p* < 0.001)-fold; p-IKKα/βlevels by 0.75 (*p* < 0.001)-fold, 0.63 (*p* < 0.001)-fold, and 0.49 (*p* < 0.001)-fold; p-NF-κB levels by 0.75 (*p* < 0.001)-fold, 0.64 (*p* < 0.001)-fold, and 0.46 (*p* < 0.001)-fold, relative to the DMSO (VC) (Figure 6E,F). Meanwhile, similar trends were observed in OAW42 cells with MGZ exposure at 0.156, 0.312 (IC_50_), and 0.625; the level of Girdin expression decreased by 0.82-fold (*p* < 0.01), 0.53 (*p* < 0.001)-fold, and 0.27 (*p* < 0.001)-fold, respectively. Likewise, at the same MBZ concentrations, p-Akt levels decreased by 0.83 (*p* < 0.05)-fold, 0.57 (*p* < 0.001)-fold, and 0.33 (*p* < 0.001)-fold; p-IKKα/β levels by 0.8 (*p* < 0.01)-fold, 0.53 (*p* < 0.001)-fold, and 0.31 (*p* < 0.001)-fold; p-NF-κB levels by 0.83 (*p* < 0.05)-fold, 0.46 (*p* < 0.001)-fold, and 0.3 (*p* < 0.001)-fold, respectively, relative to the DMSO (VC) (Figure 6G,H). These results indicate that MBZ substantially exerts its anticancer effects on OVCAR3 and OAW42 cells through a novel mechanism of action through the Girdin-mediated Akt/IKKα/β/NF-κB signaling axis.

## 4. Discussion

Ovarian cancer (OC) continues to be the most common cause of mortality among gynecologic cancers, highlighting the urgent need for novel therapeutic approaches, especially in light of drug resistance and late-stage diagnosis. A new drug regime seems to be the need of the hour. Mebendazole (MBZ), a benzimidazole derivative traditionally used as an anti-helminthic drug, has been repurposed as an anticancer agent in various cancers such as lung, breast, pancreatic, gastric, and colorectal cancer [6]. However, its effect on OC remains obscure. In this study, we selected MBZ to decipher its anticancer mechanism against OC cell lines. Both MBZ and Cisplatin inhibited cell proliferation in a dose-dependent manner in both OVCAR3 and OAW42 ovarian cancer cell lines. With increasing drug concentrations, cell proliferation decreased. The IC_50_ values of MBZ were significantly lower than those of cisplatin for both cell lines. For OVCAR3, MBZ is approximately four times more potent, while for OAW42, MBZ is about three times more potent. This indicates that MBZ is more potent than cisplatin in inhibiting cell proliferation. Among the two cell lines, OAW42 exhibited higher sensitivity to both drugs, as reflected by the lower IC_50_ values compared to OVCAR3. These findings suggest that MBZ may have a higher potency than Cisplatin in these ovarian cancer cell lines, supporting the findings from earlier studies on MBZ’s dose-dependent anti-proliferative effects [18].

Tumor cell proliferation is closely associated with an increased migratory and invasive phenotype, a hallmark of metastasis. The high metastatic potential of ovarian tumor cells, which migrate along the peritoneal cavity, significantly contributes to poor survival outcomes; through a sequential cascade of migration, invasion, and colony formation that drive metastasis, the ability of OC cells to establish colonies further complicates disease treatment [36]. Scratch wound and 3D spheroid invasion assays revealed that MBZ was able to suppress migration and invasion in OVCAR3 and OAW42 cells in a dose-dependent manner. Furthermore, an evaluation of the colony-forming potential of OVCAR3 and OAW42 cells upon MBZ treatment demonstrated that MBZ can significantly reduce colony formation in both cell lines, conclusively validating its anti-metastatic properties observed previously in gastric cancer [30] and non-small lung cancer cell lines [37].

Cancer metastasis involves several intertwined processes, including cytoskeletal rearrangements, extracellular matrix (ECM) degradation, epithelial-to-mesenchymal transition (EMT), and various signaling pathways. Cytoskeletal remodeling, particularly of actin filaments and microtubules, enables cancer cells to adapt their shape and mobility, enhancing their metastatic potential [38,39]. MBZ is well documented to inhibit tubulin polymerization in several cancer cells [9]. Herein, flow cytometry and confocal microscopy showed that MBZ blocked actin polymerization, resulting in the disruption of actin cytoskeleton organization in both the OC cell lines, suggesting an additional mechanism of cytoskeletal disruption that contributes to its ability to impair cell migration and invasion.

Cancer cells with stem-like properties, or cancer stem cells (CSCs), contribute to aggressive tumorigenicity, as they can rapidly proliferate and establish new tumors, resulting in metastasis and chemo-resistance [40]. They express markers associated with normal stem cells (e.g., CD44) and exhibit high plasticity. Our results showed that MBZ significantly down-regulated cancer stemness markers, including CD24, CD44, and EpCAM, in both OVCAR3 and OAW42 cells, but the effects were more pronounced in OVCAR3 cells. However, EpCAM expression exhibited a more complex pattern, with an initial increase at lower MBZ concentrations, eventually decreasing at higher concentrations in OAW42 cells. These results demonstrated that MBZ may reduce stemness properties through multiple mechanisms, eventually down-regulating CSC markers.

ECM degradation is another essential process that allows cancer cells to invade surrounding tissues and migrate to distant sites. This degradation is facilitated by matrix metalloproteinases (MMPs), particularly MMP-9, which play a crucial role in enhancing the metastasis behavior of OC cells [41]. Gelatin zymography was performed for the assessment of MMP-9 activity, showing that MBZ treatment significantly inhibited MMP-9 secretion and gelatinolytic activity in a dose-dependent manner in both OVCAR3 and OAW42 cells. Additionally, EMT is a process in which epithelial cells lose their cell–cell adhesion properties and acquire mesenchymal traits, which enhance their migratory and invasive capacities [42]. Herein, Western blot analysis of EMT-related markers showed that MBZ significantly lowered the expression level of MMP-9, N-cadherin, and vimentin while increasing E-cadherin expression. Our findings substantiate previous studies of MBZ in oral carcinoma cell lines, suggesting that MBZ interferes with the metastatic process by preventing cells from detaching, migrating, and invading, which are necessary steps for colonization at distant sites [43].

Moreover, the disruption of the cytoskeleton, which is integral to cell division, can induce cell cycle arrest [44]. Our results revealed that MBZ induced G2/M cell cycle arrest in OVCAR3 and OAW42 cells, as evidenced by a decrease in gradatim in G0/G1, and increased G2/M phase populations were analyzed using a flow cytometer. In addition, Western blot analysis further confirmed that MBZ modulates cell cycle regulators, reducing Cyclin B1 and Cdc25C levels while increasing Wee1 expression dose-dependently. The observed effects align with earlier work demonstrating the efficacy of MBZ in arresting cancer cells at the G2/M phase and markedly lowering the expression level of Cyclin B1 at 48 h of MBZ treatment in TNBC. Cell cycle arrest acts as a crucial link between mitochondrial dysfunction and apoptosis. Our study demonstrated that MBZ significantly induced a disruption of the mitochondrial membrane potential (ΔΨ_m_) in a dose-dependent manner, and its effects were more pronounced in OAW42 cells than in OVCAR3 cells. This mitochondrial membrane destabilization impairs energy production and promotes oxidative stress, triggering apoptotic pathways [45]. Herein, flow cytometry analysis using Annexin V/PI staining demonstrated an increase in early and late apoptosis in MBZ-treated cells and Western blot analysis showed elevated levels of pro-apoptotic proteins, including Bax, cytochrome C, cleaved-PARP, and cleaved caspases-3, -7, and -9, alongside decreased anti-apoptotic Bcl2 expression. Our results underscore MBZ’s ability to induce apoptosis through mitochondrial disruption, aligning with previous findings stating that MBZ induces apoptosis and modulates intrinsic apoptotic axis-related proteins through mitochondrial membrane potential disruption [46].

Finally, we investigated the influence of MBZ on NF-κB signaling, which is known to promote proliferation, metastasis, chemoresistance, and immune evasion in ovarian cancer [47,48]. In this study, we found a significant decrease in the nucleus-to-cytoplasm mean fluorescence intensity (MFI) ratio, indicating reduced p65-p50 dimer translocation to the nucleus in the immunofluorescence analysis and an overall downregulation of p-NF-κB expression in OVCAR3 and OAW42 cells. These results suggest that MBZ may influence NF-κB signaling, potentially leading to anti-metastatic and pro-apoptotic effects. Previous studies have demonstrated that MBZ suppresses the PI3K/Akt and NF-κB pathways, both of which are pivotal for cancer cell proliferation, survival, and metastasis [9,49]. However, the effects of MBZ on the expression of Girdin, an enhancer of Akt phosphorylation, have not yet been explored in cancer cells. Therefore, targeting Girdin with MBZ would represent a novel approach. Henceforth, considering this, we explored MBZ’s impact on Girdin and its associated anticancer signaling cascade in OC cell lines. By conducting Western blot analysis of Girdin, p-Akt, p-IKKα/β, and p-NFκB, we discovered that MBZ treatment significantly down-regulates these molecules in a dose-dependent manner in OVCAR3 and OAW42 cells, implying anticancer effects through a novel mechanism of action via the Girdin-mediated Akt/IKKα/β/NF-κB signaling axis.

## 5. Conclusions 

In conclusion, our study demonstrates that MBZ exerts potent anticancer effects on ovarian cancer cells by targeting multiple cellular processes, such as inhibiting cell proliferation, migration, and invasion and inducing apoptosis. MBZ also triggers G2/M cell cycle arrest, down-regulates cancer stem cell markers, and exerts its effects via the Girdin-driven Akt/IKKα/β/NF-κB signaling axis, representing a novel mechanism of action. These findings highlight MBZ’s potential as a repurposed therapy for ovarian cancer, either alone or in combination with platinum-based drugs or PARP inhibitors, to enhance efficacy and overcome resistance. Its low toxicity profile supports clinical potential, but further trials are needed to validate its safety, optimize dosing, and explore its targeting of the Girdin-mediated pathway.

## Figures and Tables

**Table 1:** Time-dependent IC_50_ values for MBZ in OVCAR3 and OAW42 cells.

TIME POINT	OVCAR3 IC_50_ * (µM)	OAW42 IC_50_ * (µM)
**24 h**	1.25	0.625
**48 h**	0.625	0.312
**72 h**	0.312	0.156

* The IC_50_ denotes the cytotoxic concentration of the MBZ necessary to kill 50% of the cell population after 24, 48, and 72 h of drug exposure.

**Table 2:** MBZ concentrations for IC_25_, IC_50_, and IC_75_ after 48 h treatment in OVCAR3 and OAW42 cells.

MBZ INHIBITORY CONCENTRATION (IC) at 48 h	OVCAR3	OAW42
**IC_25_** * (µM)	0.312	0.156
**IC_50_^#^ (µM)**	0.625	0.312
**IC_75_^$^ (µM)**	1.25	0.625

The *, ^#^, and ^$^ denote the cytotoxic concentration of the MBZ necessary to kill 25% (IC_25_), 50% (IC_50_), and 75% (IC_75_) of the cell population after 48 h of drug exposure.

## Data Availability

All data presented in the article is available with the author and can be given upon reasonable request.

## Supplementary Materials

The following supporting information can be downloaded at: https://www.mdpi.com/article/10.3390/cells14020113/s1, Figure S1: Cisplatin impedes cell proliferation of OVCAR3 and OAW42 cells in a dose-dependent manner. Cells were treated with Cisplatin at varying concentrations (0.156, 0.312, 0.625, 1.25 to 2.5 μM) and DMSO-vehicle Control (VC) for 48 h. Cell proliferation was assessed using the MTT assay. The half-maximal inhibitory concentration (IC_50_) of Cisplatin was determined for each time point, and a quantitative plot was generated using GraphPad Prism software. Data represent the mean ± standard error of the mean of two independent experiments in triplicate.
