# Supplementary material for: Mebendazole Exerts Anticancer Activity in Ovarian Cancer Cell Lines via Novel Girdin-Mediated AKT/IKKα/β/NF-κB Signaling Axis
Source: Cells. 2025 Jan 14;14(2):113. doi: 10.3390/cells14020113 (PMC11763501; doi:10.3390/cells14020113)
Supplement: Supplementary file 1 [file cells-14-00113-s001.zip › cells-3399466-supplementary.pdf]

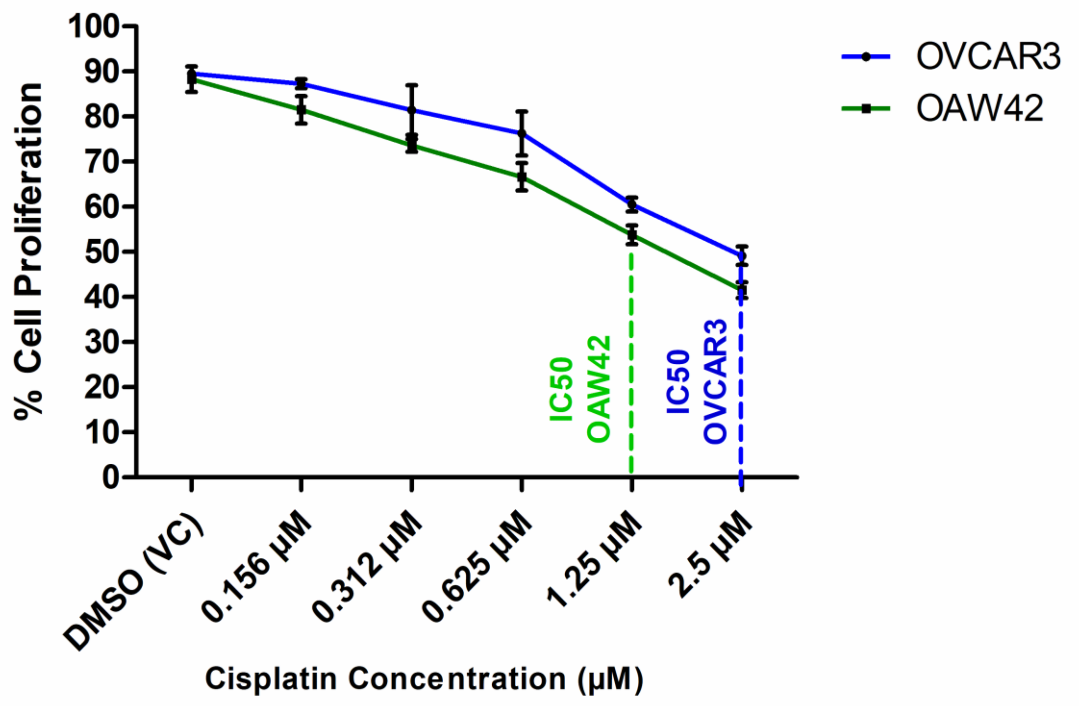

Supplementary Figure S1. Cisplatin impedes cell proliferation of OVCAR3 and OAW42 cells in a dose-dependent manner. Cells were treated with Cisplatin at varying concentrations (0.156, 0.312, 0.625, 1.25 to 2.5 µM) and DMSO-vehicle Control (VC) for 48 hours. Cell proliferation was assessed using the MTT assay. The half-maximal inhibitory concentration (IC_{50}) of Cisplatin was determined for each time point, and a quantitative plot was generated using GraphPad Prism software. Data represent the mean ± standard error of the mean of two independent experiments in triplicate
